# Risk assessment and evaluation of China’s policy to prevent COVID-19 cases imported by plane

**DOI:** 10.1371/journal.pntd.0008908

**Published:** 2020-12-07

**Authors:** Jinhua Pan, Jie Tian, Haiyan Xiong, Zhixi Liu, Ye Yao, Yesheng Wang, Wenlong Zhu, Yue Wang, Weibing Wang

**Affiliations:** 1 Department of Epidemiology, School of Public Health, Fudan University, Shanghai, China; 2 Key Laboratory of Public Health Safety of Ministry of Education, Fudan University, Shanghai, China; 3 Department of Biostatics, School of Public Health, Fudan University, Shanghai, China; University of Oxford, UNITED KINGDOM

## Abstract

As of October 5, 2020, China has reported 2,921 cases imported from overseas. Assessing the effectiveness of China's current policies on imported cases abroad is very important for China and other countries that are facing or will face overseas imported cases. In April, we used a susceptible-exposed-infectious-recovered metapopulation model to simulate the epidemic in seven foreign countries, China and the three Chinese key cities. Based on the model outside China, we estimated the proportion of people in incubation period and calculated the risk indexes for Chinese cities through analyzing aviation traffic data from these countries. Based on the model in China and the three key cities, we collected information on control measures and quantified the effectiveness of implementing the current policies at different times and intensities. Our model results showed that Shanghai, Beijing, Qingdao, Guangzhou, and Tianjin have the top five risk indexes. As of April 20, 2020, under current measures, the number of confirmed cases could be reduced by 99% compared with no air traffic restrictions and isolation measures; the reduction could be 93% with isolation of passengers only from key countries. If the current policy were postponed for 7, 10, or 20 days, the increase in the number of confirmed cases would be 1,329, 5,524, and 779,245 respectively, which is 2^.^1, 5^.^7, and 662^.^9 times the number of confirmed cases under current measures. Our research indicates that the importation control measures currently taken by China were implemented at an appropriate time to prevent the epidemic spreading and have achieved relatively good control results. However, it is necessary to remain vigilant; otherwise, another outbreak peak could occur.

## Author summary

The epidemic of the COVID-19 has greatly affected the lives of people all over the world. Before the vaccine has been widely covered, we can only reduce the transmissibility of the virus through the implementation of strong non-pharmaceutical interventions. In response to air passengers who may be carrying the SARS-CoV-2, the Chinese plan is a comprehensive measure of reduction, centralized isolation, and completely testing. We conducted a retrospective assessment of the time and intensity of the implementation of China's policy in order to provide data support for the normal implementation of future measures. The model results indicate that the measures currently taken by China were implemented at an appropriate time to prevent the epidemic spreading and have achieved relatively good effect. This means that these measures need to be strictly implemented for a period of time as the global epidemic situation is still serious. Although our model refers to the seven countries with more severe epidemics in April, the assessment method can be applied to assess the importation risks brought by other countries.

## Introduction

Since coronavirus disease 2019 (COVID-19) broke out in Wuhan, Hubei Province, the Chinese government took precautionary measures and limited the spread of the disease in the population, therefore the epidemic in China has been under control since March[1,2]. However, it has rapidly spread globally, involving 212 countries and regions[3]. With the global pandemic of COVID-19, the risks of importation from overseas in China are increasing. As of October 5, totally 2,921 confirmed cases imported from overseas have been reported in Mainland China[4], which have become the key factor in the increase in the number of daily confirmed cases there. Assuredly, with the prevalence of COVID-19 worldwide, the pressure to detect and control imported cases is also increasing.

To cope with these increasing risks, the Civil Aviation Administration of China (CAAC) has issued various notices to control the number of international passenger flights and limit the number of arriving passengers[5]. On March 29, 2020, the CAAC issued a new regulation: each domestic and foreign airline can maintain only one route and not more than one flight per week[6]. In addition to firm restrictions on the number of flights, the CAAC requires that airlines should ensure that the occupancy rate of flights both arriving in and departing from China does not exceed 75%. Relevant quarantine measures for inbound passengers were issued by provincial governments. Since March 23, 2020, most provinces have begun to change quarantine measures, from the original home or centralized quarantine for passengers from key regions to mandatory centralized quarantine for all inbound passengers, with implementation of testing via nucleic acid detection.

Not only China, the high levels of COVID-19 worldwide has put all countries at risk of importation[7]. The Chinese government had made several efforts to control the imported COVID-19 epidemic. This study fitted a dynamic model of SARS-CoV-2 China and the seven countries with the most widely spread of COVID-19 in March worldwide. We use the dynamic model of SARS-CoV-2 in the seven countries combined with flight data to estimate risk index for 27 cities with the most international traffic in China. We use the dynamic model of China and the three key cities to evaluate and quantify the comprehensive measures taken by Chinese government to prevent importation and spread of COVID-19, including the timing and intensity of their implementation. We hope that these analyses can provide theoretical support for the normalization of control measures in China and for other countries to cope with import COVID-19 risk abroad.

## Materials and methods

### Selection of countries and cities

In March, we found that seven countries outside of China had severe epidemics of COVID-19. They are: Britain, France, Germany, Iran, Italy, Spain, and South Korea. Therefore, we chose these seven countries to build dynamic models to fit their epidemic trends. By analyzing the flight data of the above seven countries, we identified the 27 Chinese cities with the greatest number of arriving passengers. We selected these 27 cities, and calculated their risk indexes to assess COVID-19 import risk.

### Data source

We derived the number of reported cases in these seven countries from the databases compiled by Johns Hopkins University[8] and the World Health Organization (WHO)[9]. We obtained information about cases in China from the National Health Commission[10] and Provincial Health Commissions of China[11]. Population density in each Chinese city was from the statistical yearbook of each provinces. We used data on daily flight bookings from January 1^st^,2020 to March 31^st^,2020 from VariFlight, which provides information about the number of daily passengers on flights from different countries to China and number of passengers on flights from China to countries overseas. Information regarding control measures was collected from the official website of CAAC and the Municipal Health Commission of Beijing, Shanghai and Guangdong.

### Model

To capture the current epidemic situation and intervention strategies, we constructed the following SEIR mathematical model. According to the characteristics of COVID-19, when a susceptible person (susceptible, S) effectively contacts a person infected with COVID-19, this person will be infected with the virus (exposed, E) and will progress to the infectious stage. The infected individual may exhibit symptomatic infection (I) or be in a subclinical state or pre-symptomatic state (U); they may receive medical care (treatment, T) when they are diagnosed, and then join the recovered group (recovery, R). It should be noted that exposed individuals become infected and have the ability to infect other susceptible individuals. The flow diagram for the model appears in S1 Fig. S1 Table presents the relevant parameters in the model. The formulas for the model are shown below, of which β_1_ represents the probability of transmission following an effective contact between infectious and exposed cases and susceptible individuals, β_2_ represents the probability of transmission following a contact between subclinical cases and susceptible individuals, q is the quarantine rate, j is the detection rate, α_1_ is the death rate, γ_1_ and d are progression rate of cases from confirmed to recovery and exposed to infection, respectively. μ_1_ and μ_2_ are ratios of subclinical and confirmed cases. n represents the number of inflows and outflows. For the 7 countries, we only model outflows to China and with no inflows. In the model of each city in China, we only model the number of inflows into China from abroad with no outflows. N is the population density of the country and city we collected. We estimated parameters by calculating the minimum sum of square (MSS) using the MATLAB R2018a (version 9.4) tool fiminsearch which is used to do unconstrained nonlinear minimization. All optimal parameter values are obtained only when the results of fiminsearch are convergent.

$\frac{dS}{dt}=-Sq-\beta_{1}S(I+E)-\beta_{2}SU\pm \mathrm{nS}/N$ (-for different countries and +for different cities in China)

$\frac{dE}{dt}=\beta_{1}S(I+E)+\beta_{2}SU-Eq-dE\pm \mathrm{nE}/N$ (-for different countries and +for different cities in China)

$\frac{dI}{dt}=dE-\mu_{1}I-{j\mu }_{2}I-\alpha_{1}I$

$\frac{dU}{dt}=\mu_{1}I-\gamma_{2}U-\alpha_{1}U$

$\frac{dT}{dt}={j\mu }_{2}I-\gamma_{1}T-\alpha_{1}T$

$\frac{dR}{dt}=\gamma_{2}U+\gamma_{1}T$

Step 1: we used the fitted SEIR model to estimate the number of people in the incubation period in 7 countries, then we calculated the proportion of people in the incubation period based on the population density of the 7 countries. Using the flight data obtained from VariFlight, we were able to determine the incubation period for reaching various cities in China by airplane. We then employed population density to calculate the risk index for cases imported from overseas for different cities in China based on the following equation:

Riskindex=proportionofpersonsinincubationperiod×populationdensity×numberofpeoplearrivingfromoverseas.

Step 2: we used the fitted SEIR model in China (included Guangzhou, Shanghai and Beijing) to evaluate different policy implemented by China and three cities in China.

We based our model on the following assumptions: The detection rate is the proportion of confirmed cases of infected people among the total population. We assumed that the detection rate would initially be relatively low in countries except for China; thereafter, the detection rate would increase at a fixed increment until it attained 70%–100% (this would vary with different countries and cities according to different testing standard seen in S2 Table). Finally, the detection rate would be relatively high with the onset of cases imported to China from overseas.

## Data and materials availability

All code and data are posted online at https://github.com/wwbgroup/Flight-data.git.

## Results

The results of this study will be presented in two parts. Step 1, we used the SEIR models fitting for seven countries’ epidemic and calculate the risk indexes of 27 Chinese cities with the most international traffic. Step 2, we used epidemic models of mainland China and the three key cities–Beijing, Shanghai, and Guangzhou to display the effect of the comprehensive measures taken by Chinese government to prevent imports spread of COVID-19.

### Epidemic models fitting for seven countries outside China

Using publicly reported data of confirmed cases and the model assumptions detailed in the previous section, we created an SEIR model to fit outbreaks in the seven countries that suffered severe epidemic of COVID-19; we smoothed the changes in the outbreak using loess regression (S2 Fig). For those seven countries, we used the SEIR model to estimate the proportion of the population in the incubation period with respect to the overall population (S3 Fig). From our model, we infer that the proportion of the population in the incubation period in Britain, Germany, France, and Iran is increasing. In other words, within a period of time, our border cities will continually face the risk of import epidemic.

### Risk index of importation of COVID-19 in major Chinese cities

**Fig 1:**
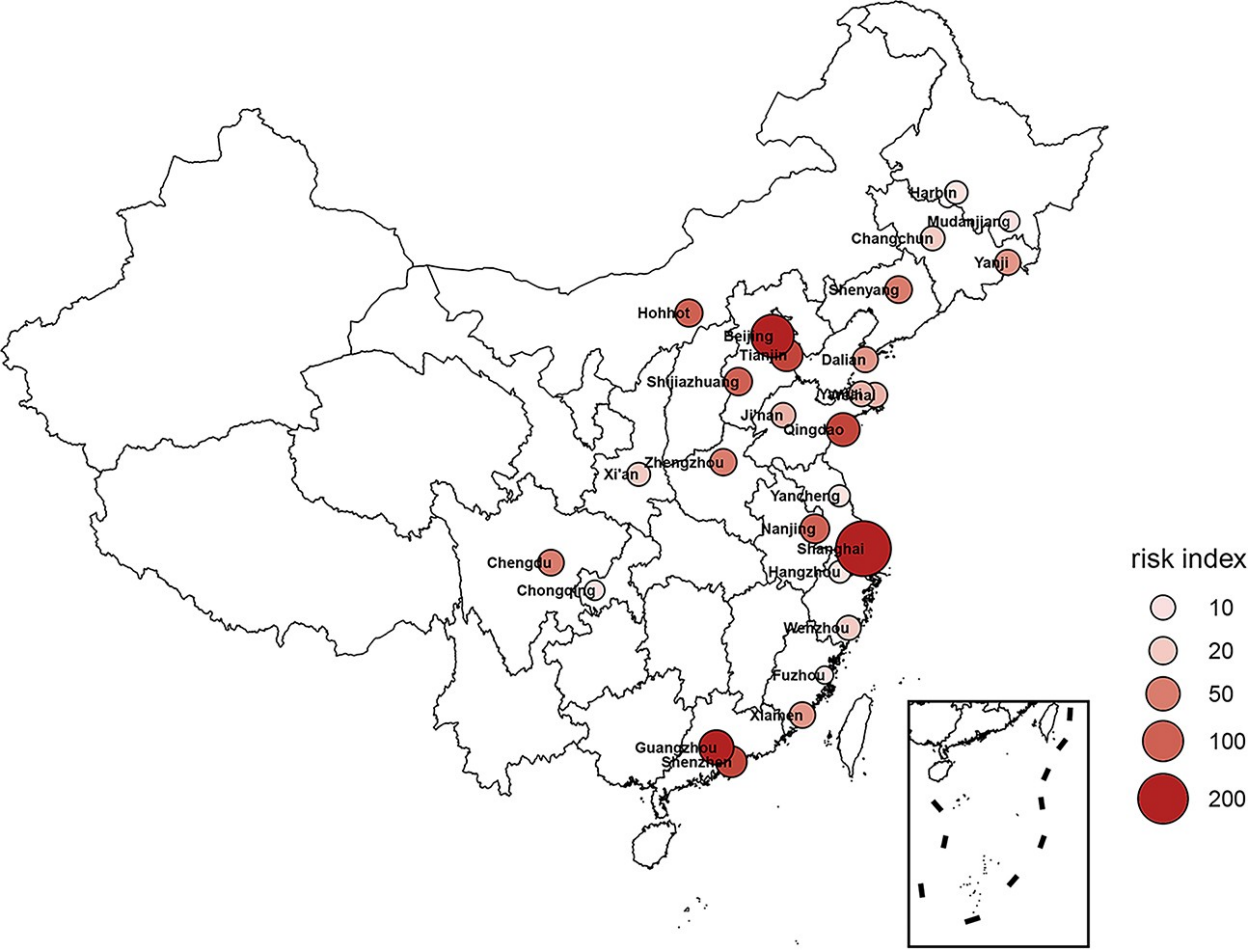
Risk index of cases imported from overseas in Chinese cities. The risk index of China’s major cities for cases imported by air traffic. The color shade indicates the level of input risk: the darker the color, the higher is the risk index.

Using the proportion of each country’s population in latency estimated by our model, combined with the flight data provided by Varilight from February 1 to March 31, 2020, we calculated the risk index for 27 Chinese cities with the most international traffic. In these cities, Shanghai, Beijing, Qingdao, Guangzhou, and Tianjin have the top five risk indexes (Fig 1). As of March 31, these five cities accounted for 59^.^68% of the total number of imported cases. The specific risk indexes of all cities are shown in Table 1.

**Table 1:** The flight data and risk index of 27 cities with the most international traffic in China.

city	Population	Flights	Passengers	risk index*
**Shanghai**	38140000	3368	358538	72396.55
**Beijing**	13130000	1697	224799	14998.43
**Guangzhou**	20048964	1206	127908	3174.672
**Qingdao**	8327247	823	80073	2225.697
**Tianjin**	8930000	184	18884	1818.254
**Shenzhen**	65215498	322	24774	1290.118
**Nanjing**	12807348	189	17703	671.0391
**Shijiazhuang**	7626659	4	679	424.4703
**Huhehaote**	3126000	18	3190	386.8727
**Shenyang**	5665385	229	26573	316.9448
**Zhengzhou**	9991942	66	6598	277.1253
**Chengdu**	11391699	402	39780	209.2977
**Xiamen**	24185149	313	22434	199.641
**Dalian**	4731568	306	21489	170.1207
**Yanji**	3146485	156	13547	157.4097
**Yantai**	5181017	86	7520	138.7009
**Weihai**	4881835	100	8229	120.954
**Ji'nan**	7282702	68	5292	117.5089
**Wenzhou**	7136392	11	820	104.1282
**Changchun**	3636770	84	8173	95.24309
**Xi'an**	9304036	144	12249	54.62944
**Harbin**	2044821	123	9561	41.57057
**Hangzhou**	4593102	153	14189	26.36298
**Yancheng**	4252554	15	794	17.01651
**Mudanjiang**	621921.2	32	2091	5.925552
**Chongqing**	3730000	117	10713	5.088581
**Fuzhou**	6467246	163	12662	1.954978

* The risk index is estimated based on proportion of persons in incubation period, population density, and number of people arriving from overseas.

**Fig 2:**
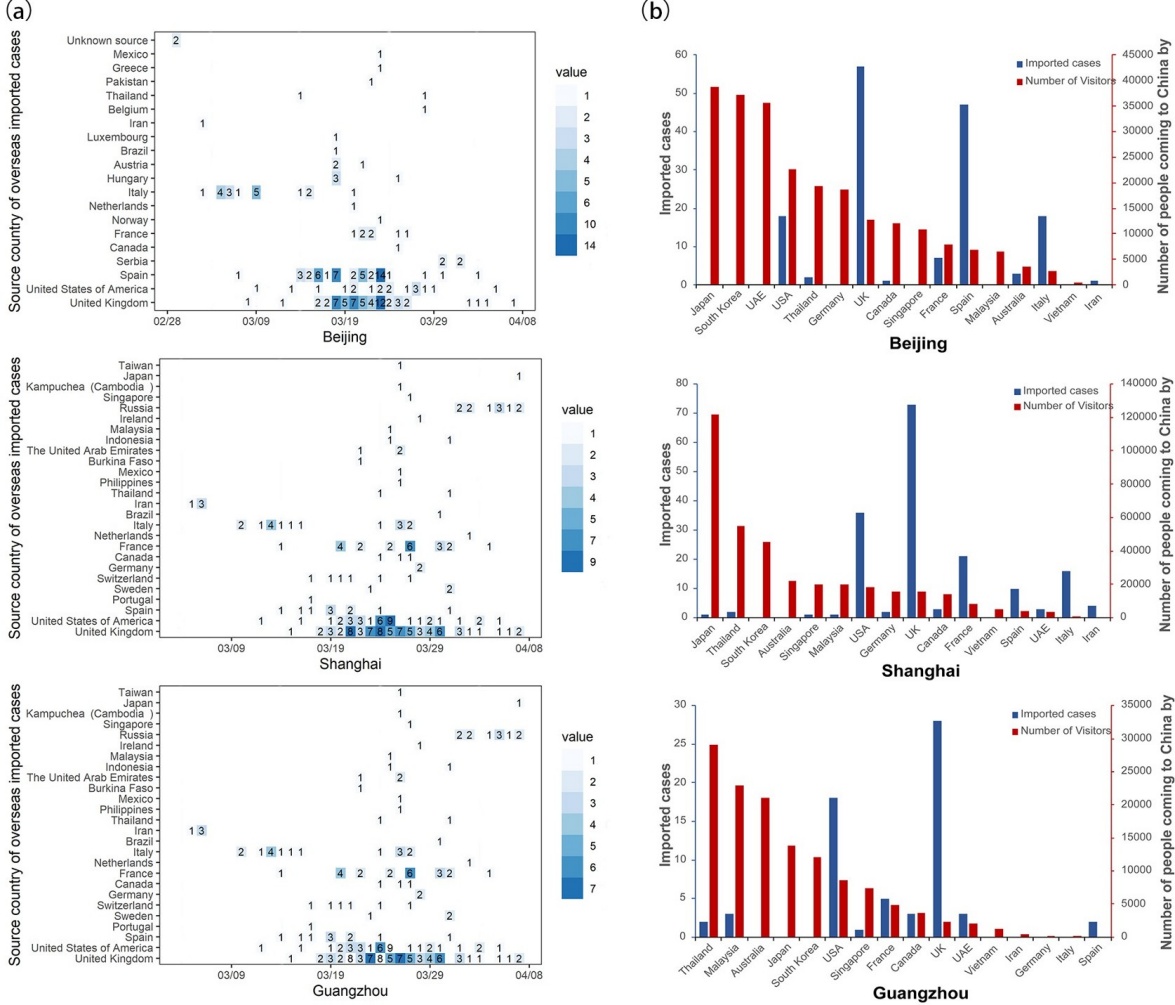
Distribution of cases imported from overseas and passenger flow to China. (a) The numbers are cases imported from a country on a particular day: the darker the color, the greater is the number of imported cases. (b) Comparison of imported cases and total passenger flow. The red bar indicates the total number of air passengers into China; the blue bar shows the number of imported cases from different countries as of April 7.

We analyzed the confirmed cases derivation of the three major cities with the most frequent international aviation exchange (Beijing, Shanghai, and Guangzhou) in China. The imported cases were mainly concentrated in mid-March; thereafter, the number of cases began decreasing following implementation of government interventions. At present, imported cases in China mainly derive from countries such as Britain, the United States, France, and Spain (Fig 2). We found that the number of inbound passengers to China from those countries was relatively small, which may reflect the large number of people in the incubation period there.

### Epidemic model fitting for China

**Fig 3:**
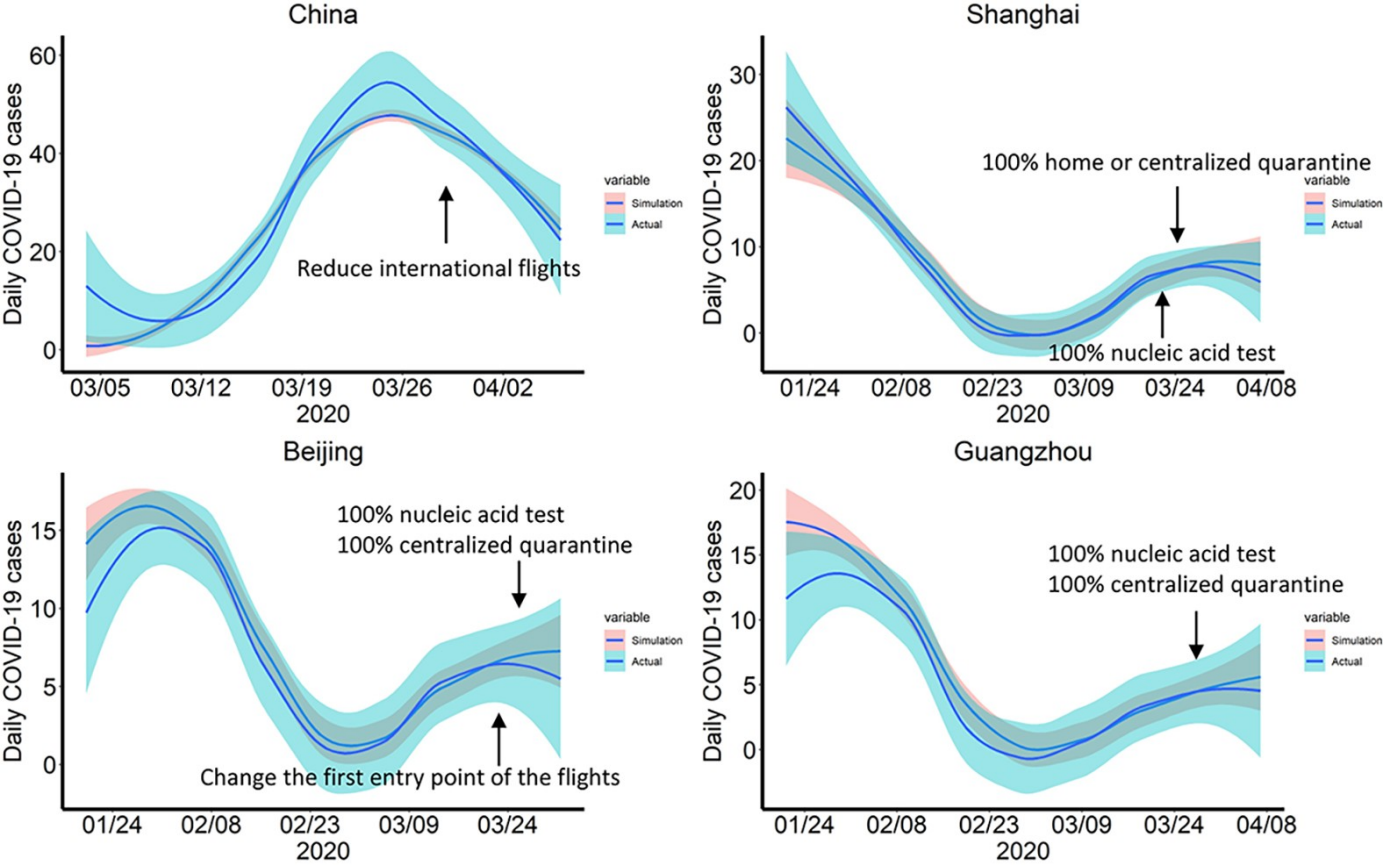
Epidemic trend in all China and in three major cities. The blue line shows the actual trend of newly imported cases; the pink line presents the fitting result with the SEIR model.

We collected the data of import confirmed cases in China and in the three key cities from March 1 to April 7, 2020 and simulated dynamic models to fit the epidemic trend. Fig 3 and S3 Table displayed that the models fit well. It is evident that the number of daily confirmed cases has dropped significantly at the end of February and rebounded in early March. Then we collected the comprehensive measures Chinese government implemented to control imported COVID-19 (S1 Text and S4 Fig), including flight and occupation rate reduction, mandatory centralized quarantine and nucleic acid test for all inbound passengers, and used fitted models to quantify and evaluate the effect of these measures.

### Effects of aviation control measures in China

**Fig 4:**
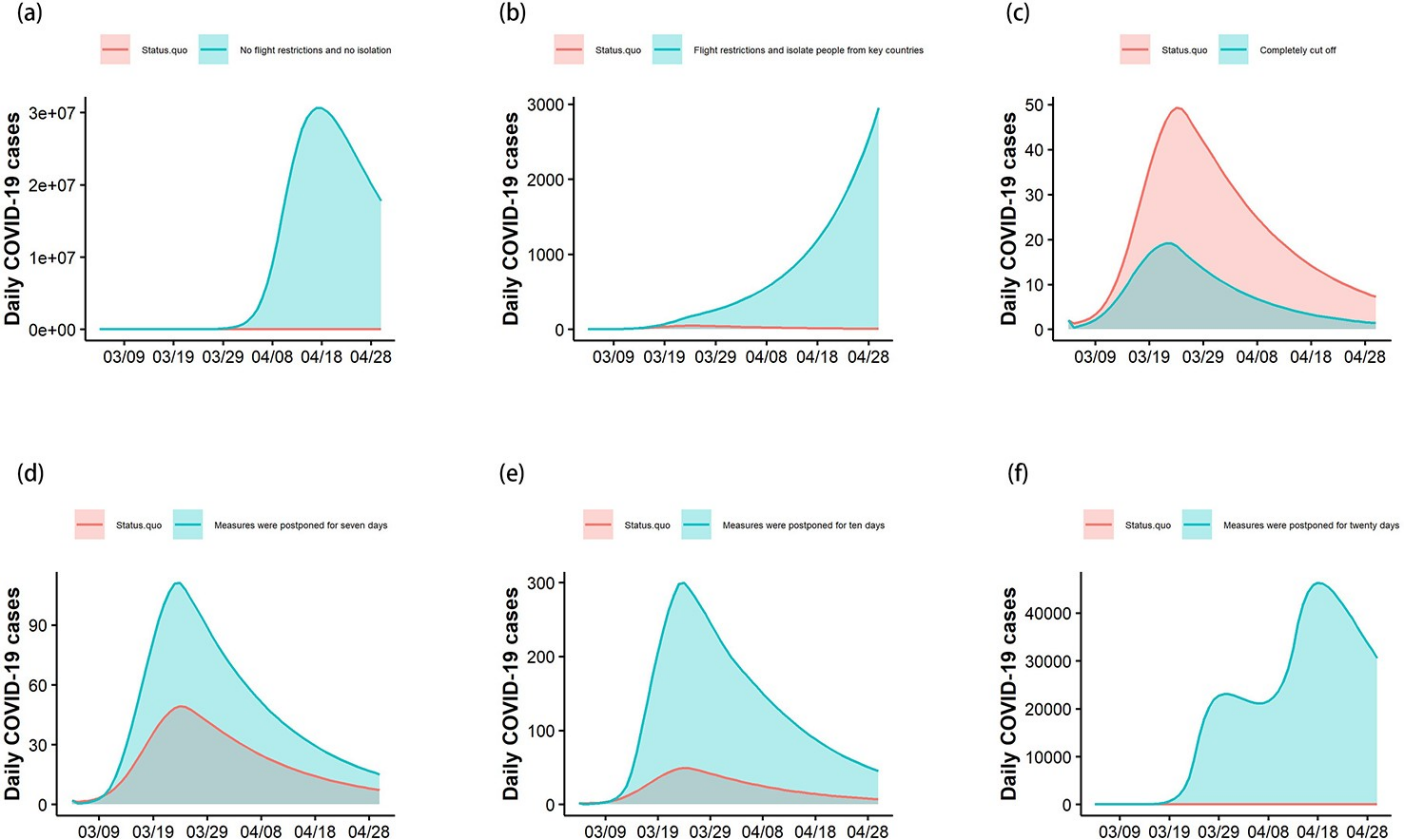
Analysis of different intervention scenarios and postponed days in China. The red curve represents the estimated number of newly confirmed cases under current measures; the blue line indicates the number of daily confirmed cases under different interventions. "Status.quo" (status quo scenario) means current status. The area between the two curves shows the difference caused under a hypothetical scenario and the actual measures. (a) No measures taken to limit the number of flights and no centralized segregation; (b) limiting the number of flights and isolating inbound passengers from countries with a severe epidemic; (c) prohibiting all entry and exit flights; (d) postponing implementation of current measures by 7 days; (e) postponing implementation of current measures by 10 days; (f) postponing implementation of current measures by 20 days.

Fig 4 shows that without measures to restrict flights and mandatory quarantine, cases imported from overseas would trigger extensive spreading of COVID-19 in China. As of April 20, current measures appear to have made a reduction of 331,354,371 confirmed cases (99% fewer cases than without measures), compared with a scenario where no measures were implemented. By controlling the number of inbound flights, if only passengers from countries with severe outbreaks were intensively quarantined for 14 days, the number of cases would increase more rapidly than with the current measures. As of April 20, existing measures could reduce 16,291 new cases (93% less cases than only quarantine passengers from severe outbreaks countries). If flights to China from overseas were completely cancelled, there would be a further reduction of 756 cases compared with current measures. Our model shows that the time of policy implementation has a great influence on the control effect. As of April 20, if the air traffic limit policy were postponed for 7 days, it would result in an increase of 1,329 confirmed cases nationwide; the increase would be 5,524 confirmed cases with a 10-day delay scenario; it would increase to 779,245 confirmed cases with a 20-day delay. Those figures are 2^.^1, 5^.^7, and 662^.^9 times, respectively, the number of confirmed cases compared with the current measures. The number of confirmed diagnoses and deaths in different implementation scenarios are shown in Table 2.

**Table 2:** Model estimates of the number of confirmed cases and deaths varying the intensity and timing of travel-related intervention scenarios, as well as the current.

Policy	China		Beijing		Shanghai		Guangzhou	
	confirmed cases	death cases	confirmed cases	death cases	confirmed cases	death cases	confirmed cases	death cases
**Current situation**	1177	47	196	8	236	9	133	5
**Completely cut off**	421	16						
**Quarantine people from key countries**	17468	699	5300	212	52012	2080	171	7
**No flight restrictions and no quarantine**	331355549	13254222						
**No acid nucleic tests and no quarantine**			12181875	487275	15123976	604959	486	19
**Postponed for 7 days**	2507	100	326	13	404	16	154	6
**Postponed for 10 days**	6701	268	760	30	1265	51	182	7
**Postponed for 20 days**	780423	31217	65940	2638	2225861	89034	312	12

* Non entries mean that the measure wasn’t run for that city. Estimated cases and deaths were through April 20.

### Effect of mandatory quarantine measures in three major cities

**Fig 5:**
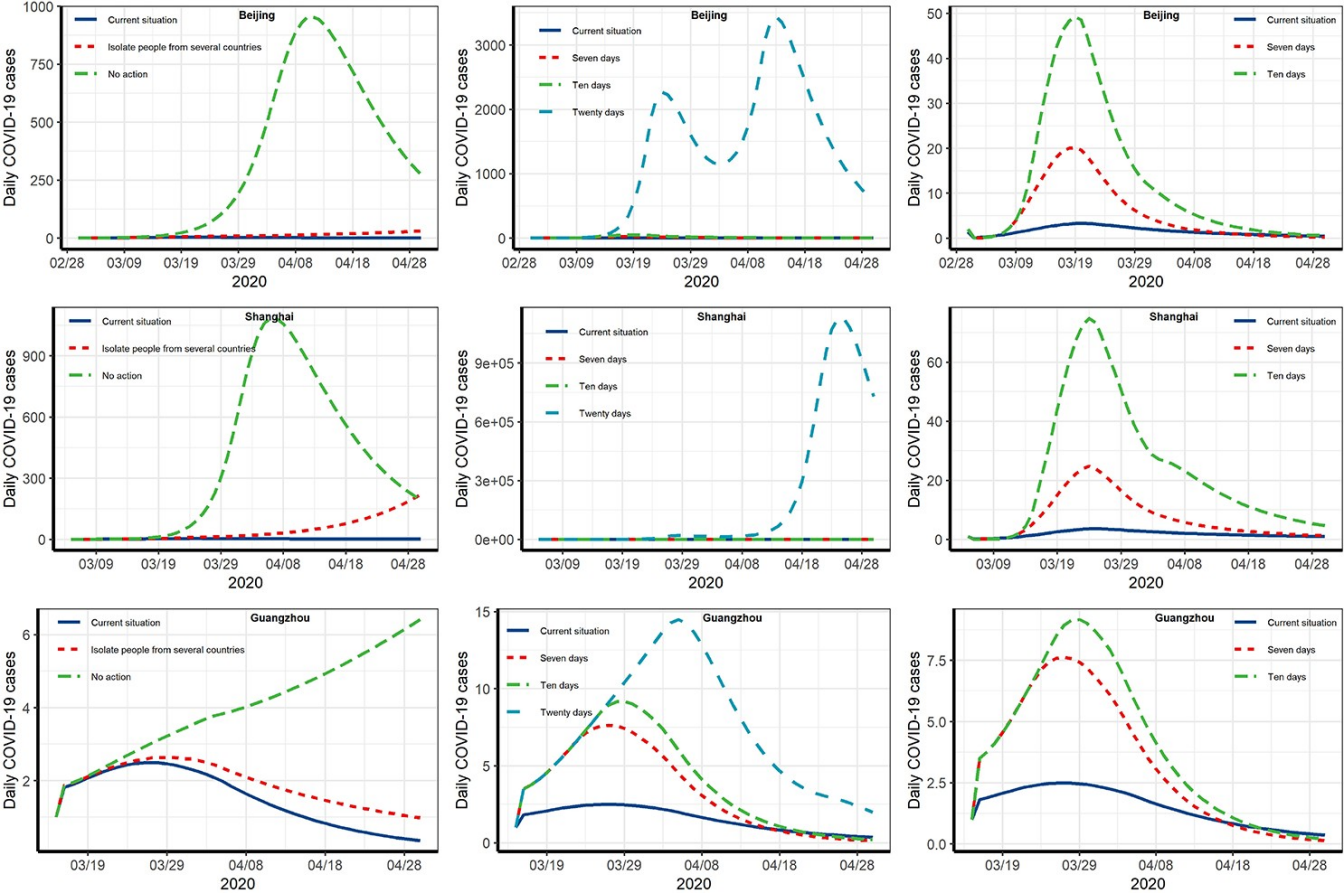
Estimated impact of current overseas control measures. Since the numbers are too large to be displayed in the same figure, we have taken the square root of the number of daily confirmed cases. The area under the curve represents the number of cases that can be reduced by implementing measures. The 7, 10, and 20 days in the figure indicate the epidemic situation if the intervention is delayed by that number of days.

Following the increase in the number of cases imported from overseas, Beijing, Shanghai, and Guangzhou, which had the most numbers of oversea travelers in China, have adopted stricter control measures for inbound travelers. We evaluated the effectiveness of the prevention and control measures adopted by those three cities (Fig 5). For Beijing, as of April 20, the model results indicate that if only passengers from high-risk countries were isolated, there would be an additional 5,104 confirmed cases (current measures reduced by 96% confirmed cases) and 205 deaths. If no measures were adopted from March 1 to April 20, the total number of confirmed cases would increase by 12,181,679 cases (current measures reduced by 99% confirmed cases); the number of deaths would increase by 489,174 (Fig 5). Our model results show that as of April 20, if only passengers from high-risk countries were quarantined, there would be an additional 51,776 confirmed cases (current measures reduced by 99% confirmed cases) and 2,079 deaths in Shanghai. If no control measures were undertaken, there would be an additional 15,123,740 confirmed cases (current measures reduced by 99% confirmed cases) and 607,316 deaths (Fig 5). According to the prediction results of our model, for Guangzhou, as of April 20, if only passengers from high-risk countries were quarantined, there would be an increase of 39 confirmed cases (22%) and two deaths. If no control measures were instigated, there would an increase of 353 confirmed cases (72%) and 14 deaths (Fig 5). These results underline the importance of remaining vigilant; otherwise, another epidemic peak will result.

We assessed the impact of the time when policy began being implemented. For Beijing, if the current measures for imported cases were postponed by 7, 10, or 20 days, the number of confirmed cases would increase by 326, 780, and 65,940, respectively; that would be 1^.^7, 3^.^9, and 336^.^5 times the number of new cases under the current policy. For Shanghai, if the isolation measures were postponed by 7, 10, or 20 days, the number of confirmed cases would increase by 168, 1,029, and 2,225,625, respectively; that would be 1^.^7, 5^.^4, and 9,423^.^9 times the number of new cases under the current policy. For Guangzhou, if the isolation measures were postponed by 7, 10, or 20 days, the number of confirmed cases would increase by 21, 50, and 179 cases, respectively; that would be 1^.^2, 1^.^4, and 2^.^3 times the number of new cases under the current policy (Fig 5). The number of confirmed diagnoses and deaths in different implementation scenarios are shown in Table 2.

## Discussion

In response to COVID-19, different countries have adopted various measures, which has led to different epidemic trends. Increase in the population in the incubation period accompanied by high-intensity aviation flow may cause a new round of virus transmission in the areas receiving these travelers. The risk index can reflect the relative magnitude of the risk of overseas imported epidemics faced by different cities, which can advise the municipal government on the implementation and relaxation of border control. A higher risk index for a city indicates that a certain number of people in the incubation period have entered the city from abroad, and strict social distancing measures need to be maintained to limit community transmission.

In terms of the intensity of the implementation of the measures, the toughest measures to completely cut off air travel can reduce the risk of the spread of the epidemic to a very low level, but complete closure will cause huge damage to the economy and trade, and is not conducive to exchanges between countries around the world. However, if no measures are taken, the virus may re-emerge and leading to a new round of pandemic, which we do not want to see. Therefore, the degree of implementation of control measures and what combination of measures can achieve a more ideal effect are the topics that this research wants to explore. China's strategy is to reduce the number of flights nationwide and implement strict border quarantine measures (quarantine + nucleic acid testing) in major cities. Our model evaluates that such a combination of strategies can also achieve a control effect similar to a complete suspension of flights. If the current measures are relaxed (quarantine only for entrants from key countries and regions), it will greatly increase the risk of an outbreak. In terms of the implementation time of the measures, it took only 17 days from the notification of the first imported case overseas by the National Health Commission on the 5th to the introduction of border control measures. The results of the model show that the speed of the policy is very timely, and delays in implementation will cause an increase in the number of infections and deaths.

Kraemer *et al*. demonstrated that asymptomatic patients may turn into confirmed patients after the quarantine period, followed by community transmission of the disease[12]. Therefore, it is important to remain vigilant; otherwise, another epidemic peak will ensue. Studies conducted in the United States and Australia[13,14] have demonstrated that travel restrictions and early detection were highly effective for preventing the importation of SARS-CoV-2 and containing the COVID-19 epidemic. Similarly, our evaluation of Chinese border control strategies indicates that the measures currently adopted were implemented at an appropriate intensity and time to achieve relatively good results in blocking imported COVID-19. Existing research indicate that the Chinese government's measures, mainly location-specific physical distancing interventions, to control local epidemics was effective[15–18]. Only when these two types of measures are implemented simultaneously can the epidemic be completely curbed. Champredon *et al*. stated that the future development of the epidemic depends on whether strict prevention and control measures are continued[19]. Kraemer *et al*. also came to the same conclusion that if the current public health interventions are relaxed, subsequent transmission may increase[20]. Relaxing or suspending current control measures may cause extensive infections and deaths of COVID-19 in China, which means, as long as the world epidemic is not over, border control measures still need to be implemented.

Certainly, the research presented here has some limitations. First, aviation data do not reflect the flow of people by other means, e.g., passengers from countries close to China may travel by other forms of transport, such as trains, cars, and ships. Second, the pre-symptomatic period has not been included in the model development and assumption of parameter values are from early literature. In addition, our model focused on import from the 7 countries with high levels of transmission at the time of analysis. We recognize that the distribution of infections will change over time, shifting the risk of importation from countries beyond our selection. But obviously, our method could be adapted to estimated importation risk over time from additional countries.

In conclusion, this study is an initial attempt to evaluate the effectiveness of the strategy the Chinese government adopted to contain the epidemic of imported COVID-19. The border control and quarantine measures promulgated by the Chinese government and municipal governments are appropriate in intensity and time. Without completely blocking traffic, the spread of the virus was controlled in a timely and effective manner, avoiding a large number of infections and deaths. Our model demonstrated that these strategies were able to effectively reduce the number of imported cases and might serve as an example for other countries with regard to preparedness and response against imported COVID-19.

## Supporting information

S1 Fig Flow diagram for the model. β1 represents the transmission ability of infection cases and exposure cases, β2 represents the transmission ability of subclinical cases, q is the quarantine rate, j is the detection rate, α1 is the death rate, and i is the immigrant number from other countries, γ1 and d are progression rate of T to R and E to I respectively. μ1 and μ2 are ratios of U and T.
(TIF)

S2 Fig Results of model fitting in seven countries. The pink line represents the result of model fitting; the blue line indicates the actual situation of the epidemic in the countries; both lines use loess regression for smoothing; the shaded area is the 95% confidence interval.
(TIF)

S3 Fig Estimates of proportion of people in the incubation period in seven countries. The lines with different colors represent the trend of the proportion of people in incubation in different countries estimated using the SEIR model.
(TIF)

S4 Fig Timeline of Chinese government aviation control measures. Aviation control measures adopted by china and three major cities. The time in the picture refers to the time when the measure starts to execute.
(TIF)

S1 Table Relevant parameters in the model. The specific values and sources of the parameters used in the model.
(DOCX)

S2 Table Detailed diagnostic criteria for COVID-19 in various countries. As of April 20, the diagnostic criteria for COVID-19 in different countries.
(DOCX)

S3 Table Statistical indicators of model fitting effect. Statistical indicators for evaluating model fitting effects.
(DOCX)

S1 Text Measures taken by the Chinese government to reduce importation risk. These measures issued by the national and municipal governments, including measures to reduce the number of people entering and measures to quarantine.
(DOCX)
