# Supplementary figures and images for: Risk assessment and evaluation of China’s policy to prevent COVID-19 cases imported by plane
Source: PLoS Negl Trop Dis. 2020 Dec 7;14(12):e0008908. doi: 10.1371/journal.pntd.0008908 (PMC7746261; doi:10.1371/journal.pntd.0008908)

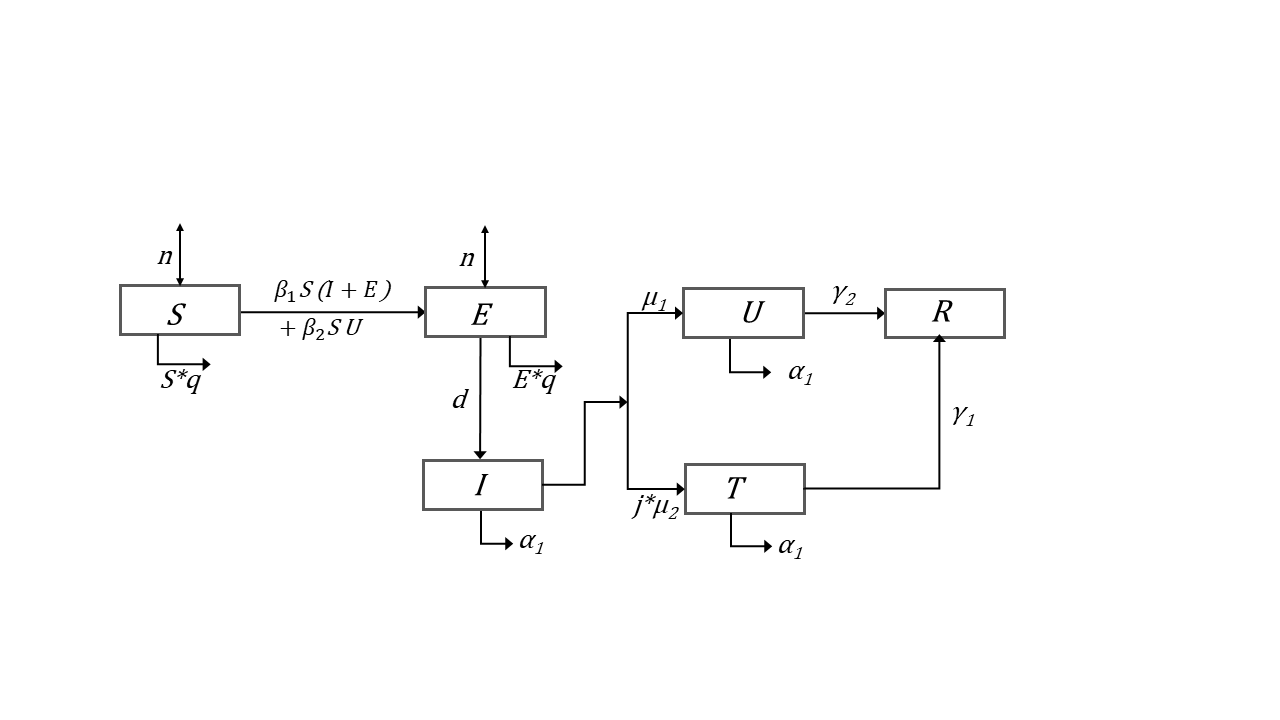

Supplement: S1 Fig — β1 represents the transmission ability of infection cases and exposure cases, β2 represents the transmission ability of subclinical cases, q is the quarantine rate, j is the detection rate, α1 is the death rate, and i is the immigrant number from other countries, γ1 and d are progression rate of T to R and E to I respectively. μ1 and μ2 are ratios of U and T. (TIF) [file pntd.0008908.s001.tif]

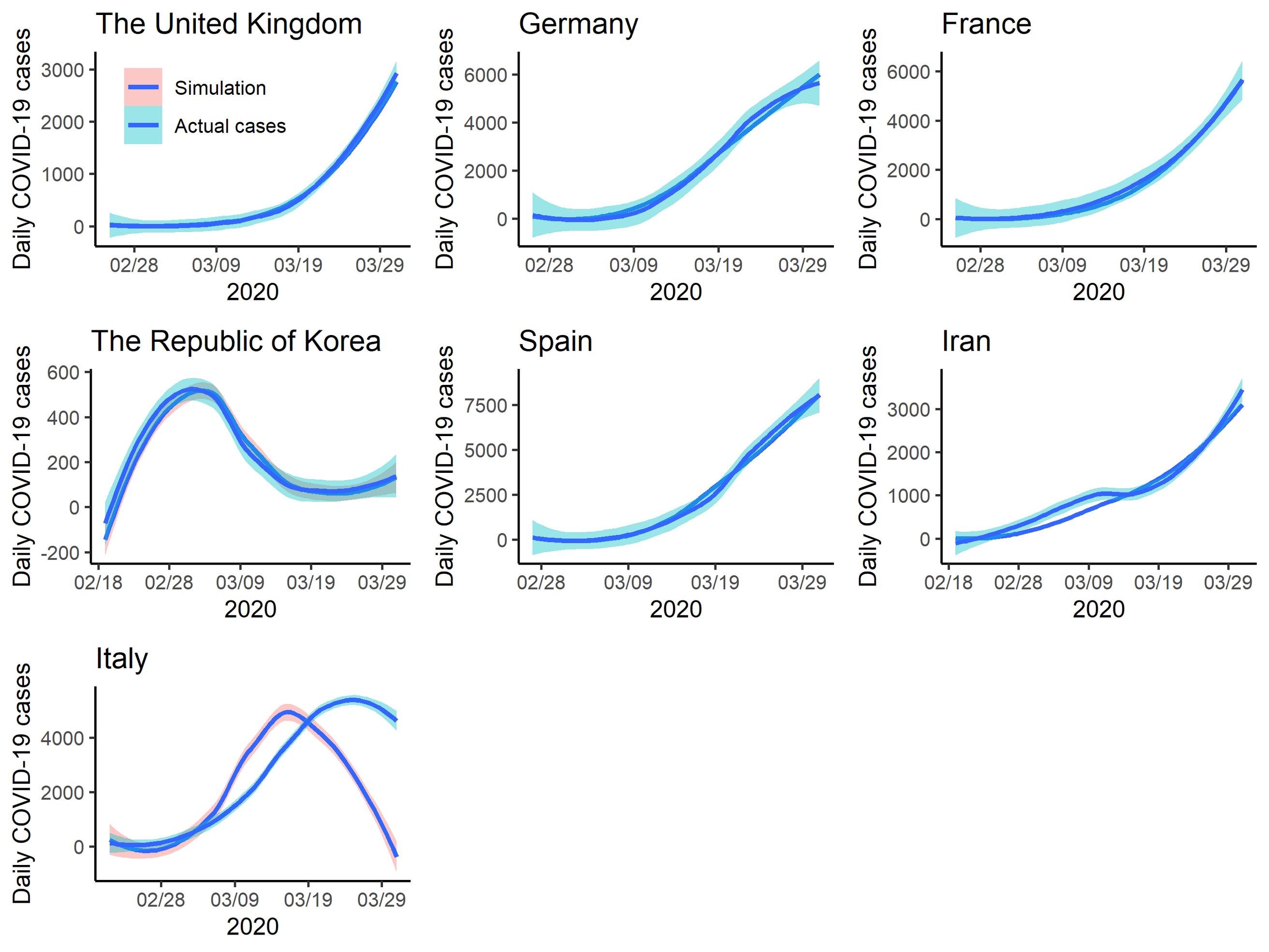

Supplement: S2 Fig — The pink line represents the result of model fitting; the blue line indicates the actual situation of the epidemic in the countries; both lines use loess regression for smoothing; the shaded area is the 95% confidence interval. (TIF) [file pntd.0008908.s002.tif]

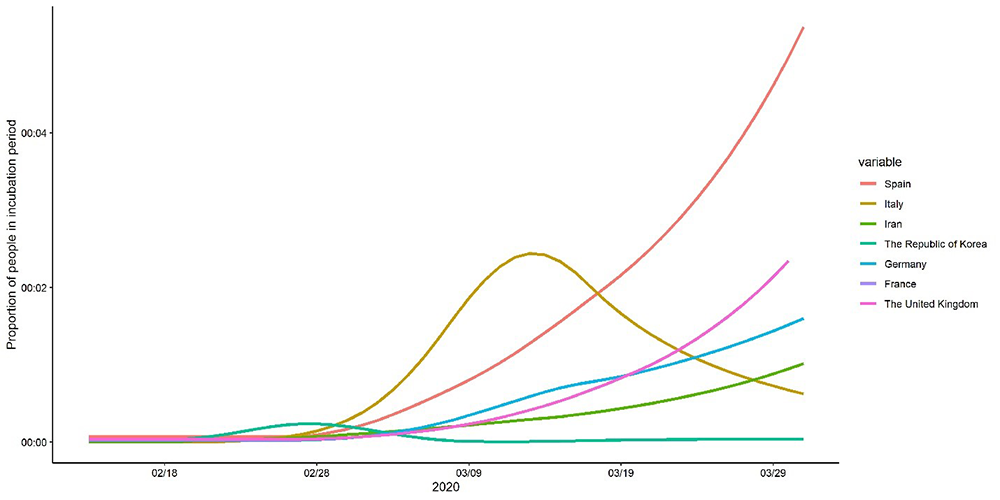

Supplement: S3 Fig — The lines with different colors represent the trend of the proportion of people in incubation in different countries estimated using the SEIR model. (TIF) [file pntd.0008908.s003.tif]

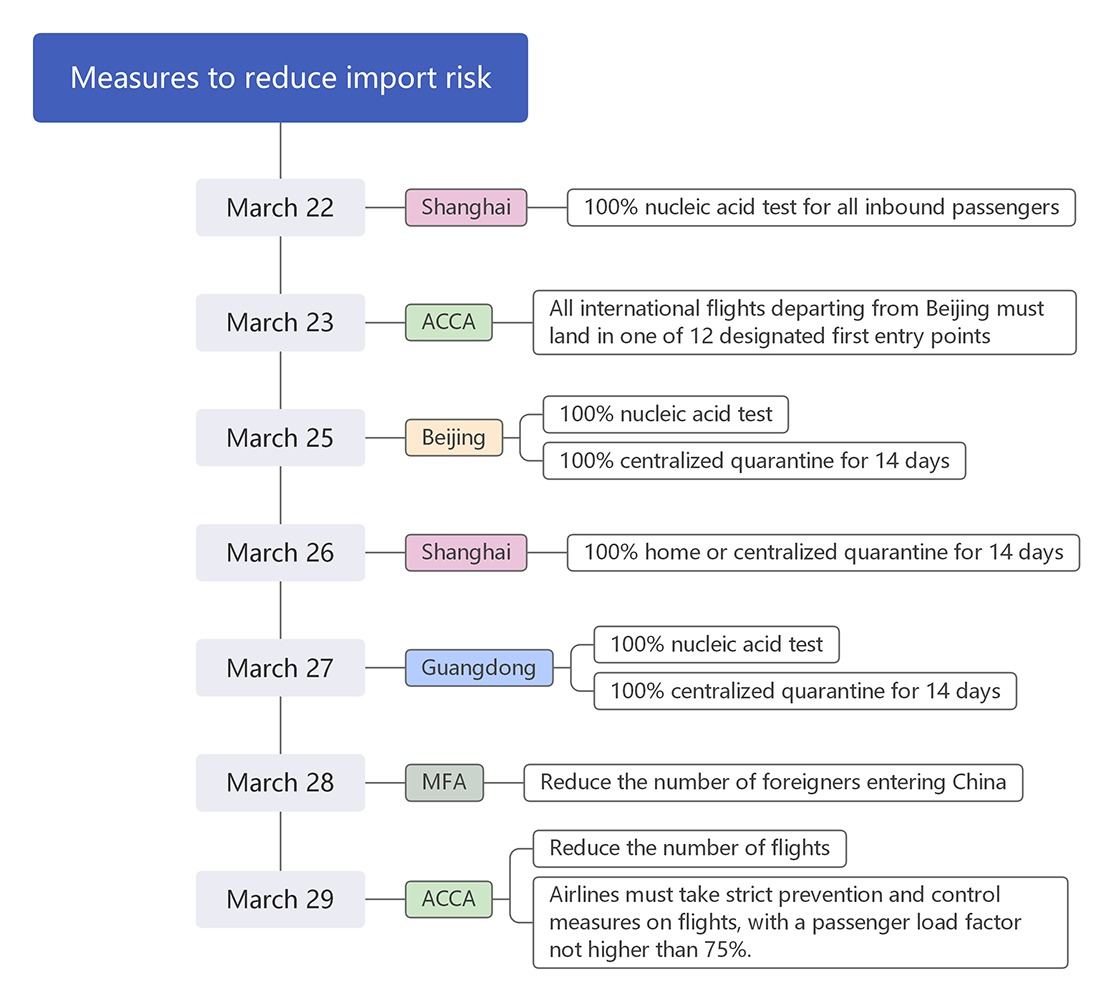

Supplement: S4 Fig — Aviation control measures adopted by china and three major cities. The time in the picture refers to the time when the measure starts to execute. (TIF) [file pntd.0008908.s004.tif]
