# Supplementary material for: Risk assessment and evaluation of China’s policy to prevent COVID-19 cases imported by plane
Source: PLoS Negl Trop Dis. 2020 Dec 7;14(12):e0008908. doi: 10.1371/journal.pntd.0008908 (PMC7746261; doi:10.1371/journal.pntd.0008908)
Supplement: S1 Table — The specific values and sources of the parameters used in the model. (DOCX) [file pntd.0008908.s005.docx]

**S1 Table. Relevant parameters in the model**

| **Parameter** | **description** | **Value** | **Source** |
| --- | --- | --- | --- |
| d | Transition rate of latent individuals to infections | 1/14 | Public data |
| γ_1_ | Transition rate from hospitalization to recovery | 1/14 | Public data |
| γ_2_ | Transition rate of subclinical infections to recovery | 1/7 | Assumed |
| λ | Transition rate of infectious individuals to quarantined infections | 1/5~1/3.800 | Public data |
| μ_1_ | The proportion of U compartment | 0. 3 | Public data |
| μ_2_ | The proportion of T compartment | 0.7 | Public data |
| q | Quarantined rate of individuals | Change with time | Assumed |
| j | detected rate of infection individuals | Change with time | Assumed |
| i | immigrated rate of susceptible individuals and exposed individuals | Change with time | Assumed |
| α_2_ | Death rate | 0.002~0.019 | Reported data |
