# Supplementary material for: Risk assessment and evaluation of China’s policy to prevent COVID-19 cases imported by plane
Source: PLoS Negl Trop Dis. 2020 Dec 7;14(12):e0008908. doi: 10.1371/journal.pntd.0008908 (PMC7746261; doi:10.1371/journal.pntd.0008908)
Supplement: S2 Table — As of April 20, the diagnostic criteria for COVID-19 in different countries. (DOCX) [file pntd.0008908.s006.docx]

**S2 Table. Detailed diagnostic criteria for COVID-19 in various countries**

| **Country** | **Criterion** | **Definition** |
| --- | --- | --- |
| South Korea | Diagnostic criteria | Patient: A person whose infectious agent is confirmed by diagnostic test criteria.  Diagnostic test criteria: virus isolation from samples, specific genetic testing. |
|  | Reporting standards | 1. Confirm the person infected with the virus through pathogen detection, whether or not clinical symptoms appear  2. Visit China (including Hong Kong and Macau) within the past 14 days + [fever (above 37.5 ℃) or respiratory symptoms (cough, sore throat, etc.)]  3. Contact the diagnosed patient within the past 14 days + [fever (above 37.5 ℃) or respiratory symptoms (cough, sore throat, etc.)]  4. Pneumonia of unknown cause requires hospitalization according to the doctor's opinion  5. In the past 14 days, visit the country of origin of Corona 19 + [fever (above 37.5 ℃) or respiratory symptoms (cough, sore throat, etc.)]  6. People suspected of being infected with Corona 19 based on doctor's opinion. |
| Japan | Reporting standards | 1. Symptomatic laboratory confirmed cases: cases with clinical symptoms (see clinical symptoms below) should be tested for virus isolation or PCR.  2. Asymptomatic cases confirmed by the laboratory: Suspected cases or asymptomatic cases under investigation should be tested for virus isolation or PCR.  3. Suspected cases, without laboratory confirmation: If a suspected new coronavirus case presents with clinical symptoms or meets the suspected standard, even if it is not confirmed by the laboratory, it must be reported to the local public health center immediately. |
|  | Clinical symptom | Clinical symptoms: The incubation period is 2-10 days, and then cold symptoms such as fever, cough and general malaise appear. Other symptoms include dyspnea or other severe symptoms, which may appear 5-14 days after the initial infection. May appear as invasive shadows seen on chest X-rays and CT. Elderly patients and individuals with new coronavirus infections that develop comorbidities are at higher risk of serious complications. |
|  | Suspected case | A) The patient has a fever or respiratory symptoms (mild to severe) and a history of close contact with a confirmed case of a new coronavirus.  B) In addition to the history of travel or residence of the new coronavirus outbreak area reported by the World Health Organization within 14 days before the onset of symptoms, the patient also developed fever (≥37.5 ° C) and acute respiratory symptoms.  C) The patient developed a fever (≥37.5 ° C), acute respiratory symptoms within 14 days before the onset of symptoms, and was in close contact with people who traveled or lived near the outbreak area of the new coronavirus reported by WHO.  D) The patient exhibits severe fever and respiratory symptoms, requiring ICU or ICU-like treatment, resulting in the inability to diagnose the patient. The doctor should treat the patient as a suspicious case of coronavirus without laboratory confirmation. |
| Malaysia | Confirmed cases | Laboratory confirmed infection 2019-nCoV |
|  | PUI | Fever or non-fever or inflammation or acute respiratory tract infection (a sudden onset of respiratory tract infection, at least one of the following: shortness of breath, cough or sore throat) and travel to or live in China 14 days before the onset of symptoms Close contact for 2019-nCoV cases. |
| Australia | Confirmed cases | Positive for SARS-CoV-2 nucleic acid test or virus identification by electron microscope or virus culture. |
|  | Suspected case | If it meets epidemiological criteria (travel to (including through) mainland China or close contacts within 14 days before onset) and clinical criteria (fever and acute respiratory infections with or without fever (eg, shortness of breath or cough)), It is classified as a suspected case. |
|  | PUI | It is recommended that clinicians should consider examining people with clinically compatible diseases in any of the following countries / regions within 14 days before the onset of symptoms: Cambodia, Hong Kong, Indonesia, Iran, Italy, Japan, Singapore, South Korea, Thailand. This recommendation does not apply to passengers transiting through the airport only in these countries. |
| Italy | Confirmed cases | Those who confirmed SARS-Cov-2 positive in the Infectious Diseases Laboratory of the Advanced Institute of Health did not consider clinical signs and symptoms. |
| France | Confirmed cases | The laboratory confirmed that people infected with sars-cov-2, whether or not they have clinical symptoms. |
| Germany | Confirmed cases | Clinical symptoms: any severe acute respiratory symptoms.  Laboratory: Positive results using at least one of the following two methods: culture positive or nucleic acid test (eg PCR) positive.  Epidemiology: contact with confirmed personnel within 14 days or have been to high-risk areas. |
| United Kingdom | Confirmed cases | The suspected patient underwent laboratory examination, and the first positive was considered as presumptive positive. Presumptive positive persons are transferred to PHE Colindale for confirmation test.  Negative results do not require further testing. |
|  | Reporting standards | Any cases that meet the testing standards for avian influenza should be reported to the local PHE health protection team.  1. From March 13th, all people with symptoms of cough and fever must be quarantined at home for 7 days. After 7 days, if symptoms persist or worsen, call 111 or call an ambulance.  2. The British government no longer tests every symptomatic person. For example, people who are isolated at home with only mild symptoms do not need to be tested. In the future, they will focus on patients with severe symptoms. |
| United States | PUI | 1. Signs / symptoms of fever or lower respiratory tract disease (eg coughing or shortness of breath) and close contact with laboratory-confirmed patients within 14 days.  2. Signs / symptoms of fever or lower respiratory tract disease (such as coughing or shortness of breath) require hospitalization and have a history of staying in the affected area within 14 days  3. Signs / symptoms of fever or lower respiratory tract disease (such as pneumonia or acute respiratory syndrome) require hospitalization, exclude other diagnoses such as influenza, and do not need to determine the source of exposure. |
| Switzerland |  | The Swedish official decided on the 12th to stop the statistics of confirmed cases of new coronary pneumonia, and the country will no longer test mild and suspected patients. |
