# Supplementary material for: Risk assessment and evaluation of China’s policy to prevent COVID-19 cases imported by plane
Source: PLoS Negl Trop Dis. 2020 Dec 7;14(12):e0008908. doi: 10.1371/journal.pntd.0008908 (PMC7746261; doi:10.1371/journal.pntd.0008908)
Supplement: S3 Table — Statistical indicators for evaluating model fitting effects. (DOCX) [file pntd.0008908.s007.docx]

**S3 Table. Statistical indicators of model fitting effect**

| Indicators | Beijing | Shanghai | Guangzhou | China |
| --- | --- | --- | --- | --- |
| t | -0.579 | -0.096 | -0.763 | 0.3328 |
| p value | 0.5634 | 0.9237 | 0.4464 | 0.7404 |
| R^2^ | 0.6361 | 0.8382 | 0.6657 | 0.7781 |
