# Supplementary material for: Risk assessment and evaluation of China’s policy to prevent COVID-19 cases imported by plane
Source: PLoS Negl Trop Dis. 2020 Dec 7;14(12):e0008908. doi: 10.1371/journal.pntd.0008908 (PMC7746261; doi:10.1371/journal.pntd.0008908)
Supplement: S1 Text — These measures issued by the national and municipal governments, including measures to reduce the number of people entering and measures to quarantine. (DOCX) [file pntd.0008908.s008.docx]

**Measures taken by the Chinese government to reduce import risk**

In response to cases imported from overseas, control measures have been undertaken in cities such as Beijing, Shanghai, and Guangzhou. Since March 23, all international passenger flights departing from Beijing have been required to land in one of 12 designated first-entry points (Tianjin, Shijiazhuang, Taiyuan, Hohhot, Shanghai Pudong, Ji’nan, Qingdao, Nanjing, Shenyang, Dalian, Zhengzhou, Xi’an)[1]. At those 12 designated first-entry points, the customs office strictly implements quarantine measures such as health declaration and temperature screening. Since March 25, all arrivals in Beijing, regardless of their destination, must accept nucleic acid testing and centralized quarantine for 14 days[2]. Shanghai has also adjusted its prevention and control policies in response to increasing risks of imported cases. Since March 22, all arrivals in Shanghai will be implemented nucleic acid testing[3]. Before March 26, Shanghai mainly imposed 14-day isolation on all arrivals who had travel history of 24 key countries. Since March 26, Shanghai has implemented 14-day health observation on all arrivals from overseas[4]. Since March 27, Guangdong Province has implemented nucleic acid testing for all arrivals from overseas (including from Hong Kong, Macao, and Taiwan and also transit passengers) to Guangdong ports. It has instituted 14-day centralized medical observation. For certain individuals who play a key role in Hong Kong and Macao, centralized medical observation is not being implemented; however, they all have to undergo nucleic acid testing.[5]

1. Announcement regarding the entry of international flights destined for Beijing from the designated first point of entry <http://www.caac.gov.cn/XXGK/XXGK/TZTG/202003/t20200322_201625.html>: Civil Aviation Adminstration of China; 2020. Available from: <http://www.caac.gov.cn/XXGK/XXGK/TZTG/202003/t20200322_201625.html>.

2. Test & Quarantine Required for All Inbound Passengers <http://wb.beijing.gov.cn/home/ztzl/kjyq/fk_yqzc/202003/t20200325_1735704.html>: Foreign Affairs Office of the People's Government of Beijing Municipality; 2020. Available from: <http://wb.beijing.gov.cn/home/ztzl/kjyq/fk_yqzc/202003/t20200325_1735704.html>.

3. Shanghai further strengthens port control <http://wsjkw.sh.gov.cn/xwfb/20200323/33243ad406964b9ea65016b56abae63e.html>: Shanghai Municipal Health Commission; 2020. Available from: <http://wsjkw.sh.gov.cn/xwfb/20200323/33243ad406964b9ea65016b56abae63e.html>.

4. For all personnel coming to Shanghai, a 14-day quarantine health observation will be implemented <http://wsjkw.sh.gov.cn/xwfb/20200326/54d26d4d76a347ffbccc187f8359ab1c.html>: Shanghai Municipal Health Commission; 2020. Available from: <http://wsjkw.sh.gov.cn/xwfb/20200326/54d26d4d76a347ffbccc187f8359ab1c.html>.

5. Notice of Guangzhou Epidemic Prevention and Control Headquarters on Further Strengthening the Prevention and Control of Importation of Overseas Epidemic Situations (No. 8) <http://www.gz.gov.cn/xw/tzgg/content/post_5746398.html>: Guangzhou Municipal People's Government; 2020. Available from: <http://www.gz.gov.cn/xw/tzgg/content/post_5746398.html>.
